# Implementation and outcomes of a brief crisis intervention for adolescents with borderline personality features: a pre-post observational study

**DOI:** 10.1186/s12888-026-07871-y

**Published:** 2026-02-06

**Authors:** Dianna R. Bartsch, Justine C. Price, Luke Tilley, Sophie C. Dahlenburg, Simon Cousins, Mohammed Usman, Sierra Magann, Cathy McLeod Everitt

**Affiliations:** 1 Borderline Personality Disorder Collaborative, Barossa Hills Fleurieu Local Health Network (BHF LHN), 1/100 Greenhill Road, Unley, South Australia 5061 Australia; 2 School of Psychology, College of Education, Behavioural and Social Sciences, Adelaide University, Adelaide City Campus East, Adelaide, South Australia 5000 Australia; 3 Child and Adolescent Mental Health Service, Women and Children’s Health Network, 72 King William Road, Adelaide, South Australia 5000 Australia

**Keywords:** Crisis, Brief intervention, Adolescents, Suicide, Borderline personality disorder, Borderline personality features, Emergency department, Perceived burdensomeness, Thwarted belongingness, Implementation

## Abstract

**Background:**

Suicide is one of the leading causes of death among young people. Borderline personality features have been identified as a risk factor for suicide-related behaviour among adolescents. Targeted brief interventions may facilitate early identification and intervention.

**Methods:**

A single-group pre-post observational study design examined implementation of a brief crisis intervention (Gold Card SA) for adolescents aged 12–17 years-of-age, delivered in outpatient hospital and community mental health settings. The intervention offered up to three weekly psychologically focussed sessions to explore goals and values, provide psychoeducation and develop a collaborative care plan. A fourth session was offered to the adolescent’s nominated support person. We examined patient-reported outcome measures collected at the first and final appointment and service outcomes (e.g., acute mental-health service utilisation comparing the 6-months pre- and postintervention). We also considered our findings in light of early implication indicators such as acceptability, appropriateness, fidelity, penetration and sustainability.

**Results:**

One-hundred and fifty-five adolescents consented for their outcomes to be evaluated (12–15-year-olds *n* = 46; 16–17-year-olds *n* = 109). Borderline features were high among adolescents attending their initial Gold Card SA appointment. The incidence of emergency department presentations in the 6-months postintervention was 90% lower relative to the 6-months beforehand (95% CI [0.05, 0.18]; *p* < .001). Adolescents who completed the intervention demonstrated a significant reduction in perceived burdensomeness (12–15 years, *d*_*av*_ = 0.31, *p* = .001; 16–17 years old, *d*_*av*_ = 0.51, *p* < .001) and psychosocial dysfunction (12–15 years, *d*_*av*_ = 0.31, *p* = .006; 16–17 years old, *d*_*av*_ = 0.31, *p =* .01). Adolescents aged 16–17 years also reported significant reduction in borderline symptom severity (*d*_*av*_ = 0.89, *p* < .001) and the likelihood of engaging in deliberate self-harm (Exp (B) = 0.86, *p* = .03) pre- and postintervention.

**Conclusions:**

A brief intervention model such as Gold Card SA, embedded within a public mental health setting, may support adolescents and their families during crisis. Screening for borderline personality features at this time may facilitate early intervention whereby at-risk adolescents are offered additional intervention via a broader stepped model of care.

**Clinical trial number:**

Not applicable.

**Supplementary Information:**

The online version contains supplementary material available at 10.1186/s12888-026-07871-y.

## Introduction

Suicide was the leading cause of death among Australian children (aged 5–17 years) in 2023 [1]. Concerningly, paediatric emergency departments worldwide have seen a significant increase in self-harm and suicidality presentations over the past decade [2]. A psychological autopsy study found that in a small sample of 15–24-year-olds who died by suicide, personality disorders were present among 30%, and there was evidence of ‘personality trait accentuation’ among 56% of the sample [3]. Research has found that among adolescents presenting to inpatient and outpatient service units, personality pathology and major depression are independent and cumulative risk factors for suicidal behaviour and lethality [4]. Furthermore, personality dysfunction has been identified as a stronger predictor of suicidal ideation and attempts than depression, age or gender in young people [5]. Such findings prompt calls for improved methods to identify, assess risk, and intervene with youth presenting in crisis particularly where borderline personality features may be present [5].

Historically, borderline personality disorder (BPD) was diagnosed after age 18 years in recognition of changes in identity development during this developmental phase [6]. However, it is now widely recognised that the emergence of symptoms may also occur in adolescence and BPD can be reliably diagnosed [7, 8]. General population estimates suggest that approximately 1–3% of youth aged under 18 years meet the diagnostic criteria for BPD [9]. Symptoms of BPD in adolescence have been associated with enduring and detrimental impacts on quality of life, interpersonal, occupational, and financial outcomes into adulthood [10]. Thus, young people presenting in crisis, who also have borderline personality features/symptoms may represent a particularly vulnerable and high-risk cohort [7, 11].

Ironically, the age that many young people start experiencing BPD symptoms is also at a time when mainstream mental health services are most reluctant to offer BPD specific supports [12]. This may be driven by fears of labelling adolescents and attracting stigma commonly associated with BPD [13]. Unfortunately, such attitudes may also hamper efforts for early intervention. O’Dwyer and colleagues [14] examined therapeutic interventions offered to young people with a BPD diagnosis in youth primary mental health care settings in Australia. They found that among (*n* = 701) youth who had a diagnosis of BPD or borderline traits documented in their first episode of care, the majority were offered cognitive behavioural therapy, supportive counselling, or interpersonal psychotherapy (IPT). The interventions offered were not associated with improvements in symptoms or functioning, prompting calls for evidence-based interventions for this cohort.

In 2013, the Australian National Health and Medical Research Counsel’s (NHMRC; 15) clinical practice for the management of BPD recommended that clinicians should assess for BPD among adolescents (aged 12–18 years) who are presenting with (1) frequent suicidal or self-harming behaviour; (2) marked emotional instability; (3) multiple co-occurring conditions; (4) non-response to established treatments; and (5) a high level of functional impairment. The guidelines also recommended that adolescents diagnosed with BPD should be referred to structured psychological therapies specifically designed for this cohort or when unavailable, onto youth mental health services. Dialectical behavioural therapy for adolescents (DBT-A) is an evidenced-based psychotherapy which has demonstrated small-to moderate effects for reducing self-harm and suicidal ideation compared to control groups [16]. However, DBT-A is resource intensive averaging between 3 and 12 months of intervention, incorporating both individual therapy and group skills training. Limited availability of highly trained therapists and funding to deliver such programs can result in substantial waiting times, and limits accessibility particularly during times of crisis [17].

Patient-initiated brief admissions in the context of acute suicidal ideation and behaviour are utilised for young people in countries such as Sweden and qualitative feedback suggests that some adolescents find this helpful for rest and recovery [18, 19]. However, inpatient admissions for adolescents are resource-intensive and have the potential to contribute to iatrogenic harm [20]. A recent review examining the effectiveness of brief interventions for young people (10–24-year-olds) with self-injurious thoughts and behaviours (SITBs) examined brief interventions defined as not exceeding 240 min (i.e. 4 × 60-minute sessions) over the past 50 years [21]. Less than half of all included studies found a positive intervention effect for at least one SITB outcome. Only ‘signs of suicide’ (SOS; a school-based psychoeducational program delivered by staff) was rated as probably efficacious, and ‘family-based crisis intervention’ (FBCI; based on cognitive-behavioural and family systems treatment models delivered in the emergency department) was considered possibly efficacious. Notably, there was no consideration of underlying BPD features and treatment efficacy within this review, and the authors called for further research into when and how brief interventions should be offered to youth presenting in crisis with suicidal thoughts and behaviours.

Recently a meta-review examined interventions for self-harm and suicidality in paediatric emergency departments (ED) and noted several common factors including follow-up after initial ED discharge, psychoeducation and safety planning, and the inclusion of family members in the intervention [2]. These elements are also key to brief interventions for adults presenting in crisis who may also have symptoms characteristic of BPD [22–24]. Huxley and colleagues [24] analysed an anonymised health service administrative dataset to examine implementation of a brief intervention as part of a broader model of stepped care in New South Wales (NSW). The analysis of pre- and postintervention assessment tools suggested that the brief intervention was associated with a large reduction in suicidal ideation (*d* = 1.01, *p* < .001) and large improvement in psychosocial functioning (*d* = 0.95, *p* < .001). Bartsch and colleagues [22] examined the statewide implementation of the same brief intervention, referred to as Gold Card SA, by analysing routinely collected service utilisation data and patient-reported outcome measures (PROMs). Across adults aged 18 years and older (*n* = 332) compared to 6-months preintervention there was a 63% reduction in the incidence of ED presentations and 65% reduction in mental-health related inpatient admissions, relative to 6-months postintervention. Furthermore, examination of pre- and postintervention PROMs found a large reduction in borderline symptom severity (BSL-23; -0.91, *p* < .001) and perceived burdensomeness (PB; *d*_*av*_ = -0.70, *p* < .001) and moderate reduction in thwarted belongingness (TB; *d*_*av*_ = -0.52, *p* < .001).

The reduction of TB and PB is a notable finding given that the Interpersonal Theory of Suicide (ITS) proposes that the desire for death is driven by feelings of PB (i.e., a belief that one’s death is worth more than their life) and TB (i.e., the lack of reciprocal caring relationships and failed attempts at interpersonal connection with others) [25, 26]. When these factors intensify, the desire for death may increase, and in the presence of lethal means, can lead to suicide. A recent study among adolescents aged 11–18 years, demonstrated that changes in suicidal ideation were mediated by changes in PB and depressive symptoms even when the young person reported elevated borderline features [27].

Teasdale and colleagues [28] described the implementation of the aforementioned brief intervention model with youth aged 12–25 years presenting to a large mental health service in NSW. Preliminary outcomes suggested a significant reduction in ED presentations (*X*^*2*^ = 25.3, *p* < .001) and inpatient bed days (*X*^*2*^ = 9.1, *p* = .01) in the 3-months following the intervention, compared to beforehand. Whilst the authors describe improved mental health among participants, outcomes relied predominantly on clinician ratings of functioning given low rates of completed adolescent self-reports postintervention, and it was unclear how many adolescents aged 12–17 years completed self-report assessments at both time points to enable comparison. In stating this, the study demonstrated that implementing this approach in a community setting with youth in acute crisis (including suicidal behaviour and/or self-harm) was feasible and acceptable, warranting further investigation.

Early intervention for personality disorder requires a shift in culture within mental health services and remains highly debated [29, 30]. Implementation of an intervention with adolescents in crisis as part of a broader model of care for BPD may be challenging in real-world settings. Proctor and colleagues [31] provide a useful taxonomy that differentiates implementation outcomes from consumer outcomes (e.g., symptomology and functioning) and service outcomes (e.g., service utilisation). Implementation outcomes include eight key factors such as acceptability of the intervention (e.g. stakeholder and patient perceptions), adoption (e.g. uptake), appropriateness (e.g. perceived fit for the given setting or patient group), feasibility (e.g. extent the intervention is successfully delivered in a setting), fidelity (e.g., degree to which it is delivered as intended), penetration (e.g. integration and reach of the intervention in the system), sustainability (e.g., extent that the practice is maintained within a service over time) and costs (e.g., the cost impact of the implementation strategy). If an intervention is not implemented well, consumer and service outcomes may be negatively impacted [31].

In the present study, we describe the implementation of Gold Card SA delivered to adolescents (under 18 years of age) presenting to mental health services in acute crisis. We will report consumer outcomes drawn from PROMs completed at initial assessment and final session. We will consider service outcomes by comparing ED presentations, inpatient admissions and bed days in the 6-months before and after the Gold Card SA intervention. We will also report early implementation indicators, where available from routinely collected information, to contextualise the findings. Specifically, we hypothesised:

1. Adolescents ($\:<18$ years of age) who complete the Gold Card SA intervention will report significant reduction in proximal risk factors for suicide (TB and PB), borderline personality features (or for older adolescents’ borderline symptom severity), and psychosocial dysfunction.
2. Additionally, 16-17-year-olds will demonstrate a significant reduction in the likelihood of engaging in self-destructive/impulsive behaviour in the last session compared to their initial appointment.
3. Adolescents who participated in the brief intervention would report a significant reduction in mental health acute service utilisation (i.e., number of emergency department presentations, inpatient admissions and bed days) in the 6-months after their final session, relative to the 6 months beforehand.

## Methods

### Study design

We utilised an uncontrolled pre-post observational study design to examine the implementation of a 4-session brief crisis intervention in routine-clinical care across mental health services in one Australian state. Reporting follows the Strengthening the Reporting of Observational Studies in Epidemiology (STROBE) guidelines for observational studies [32]. In addition, relevant elements from the Standards of for Reporting Implementation Studies (STaRI) guidelines were used to describe the intervention, its implementation and associated indicators [33].

### Implementation context

The Borderline Personality Disorder Collaborative (BPD Co) was established in 2019 with the mandate to implement a statewide model of care that aimed to improve access to services for people with borderline features or a diagnosis of BPD [34]. The model of care proposed the establishment of brief intervention clinics for people presenting to services in crisis across all ten Local Health Networks (LHNs). Child and Adolescent Mental Health Services (CAMHS) offer community-based mental health services to children up to age 16 years of age. The Women’s and Children’s Hospital (WCH) provides emergency and inpatient mental health services to young people up to the age of 18 years of age. In most LHNs, adolescents aged 16 years and older can access tertiary community mental health services via the adult teams, many of which employ dedicated youth mental health clinicians.

The BPD Co statewide model of care highlighted the need for clear and consistent responses to young people presenting in crisis and improved approaches to the diagnosis of BPD [34]. An early intervention response was proposed in which adolescents presenting with self-harm, relational distress, and risk would be offered brief interventions, via Gold Card SA. Details of the implementation strategy are available in supplementary information (see Table S1).

### Intervention

**Fig. 1:**
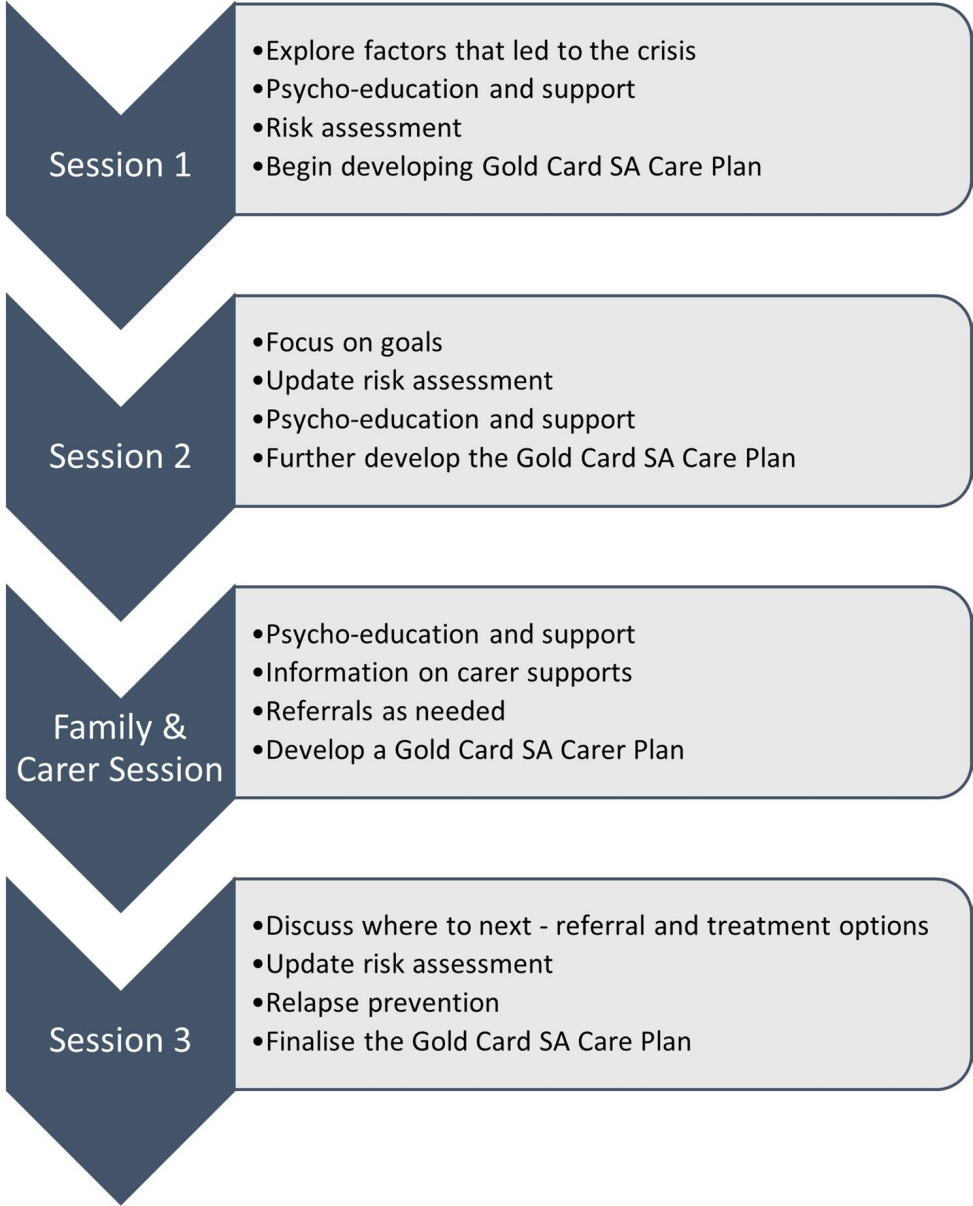
Gold Card SA session outline

Locally branded as Gold Card SA, the brief crisis intervention in the current study was developed and manualised by Project AIR [35] and is available for download on the University of Wollongong website (https://www.uow.edu.au/project-air/resources/treatment-guidelines). Participants are offered three structured psychologically focussed weekly sessions proximal to an acute crisis and an additional fourth session is offered to a support person of the young person’s choosing. The treatment guidelines are based on a relational treatment model which conceptualises a person’s difficulties as stemming from problematic relationship patterns (both intrapersonal and interpersonal) over time. An outline of session content is detailed in Fig. 1.

Gold Card SA business rules recommend that the intervention is offered to young people if they have experienced a recent mental health crisis and one or more of the following symptoms (1) suicidal thoughts or plans and/or recent episodes of self-harm or suicide attempts; (2) fluctuating intense emotions and high levels of distress; (3) problems with identity and sense of self; (4) impulsive and/or self-destructive behaviour; (5) persistent instability in relationships; (6) a diagnosis of BPD or borderline personality features. The intervention was not recommended for people with (1) a high level of risk requiring urgent follow-up; (2) people presenting with acute psychosis or affective disorder; or (3) where the primary presentation was due to alcohol and/or drug intoxication. However, it is noted that these criteria may have been revised to suit local requirements of implementation sites. Delivered within a broader statewide stepped model of care, it is expected that where participants meet criteria for BPD and if clinically indicated, that they are referred onto another level of care.

### Patient-reported outcome measures

In addition to participant demographics such as age, gender, ethnicity, and diagnosis at intake and discharge, we will examine PROMs that were embedded in the assessment package at initial assessment and after the final session.

### Suicide-related cognitions

**The Interpersonal Needs Questionnaire (INQ-15; **25**).** This 15-item measure was utilised to examine PB and TB which are proximal risk factors for suicide. Several studies have suggested that the INQ-15 may need to be adapted for youth. In particular, Hill and colleagues [36] investigated the factor structure of the tool among *n* = 593 adolescents recruited from outpatient and inpatient settings, and found that the factor structure of the scale was improved by removing three items (i.e., These days, I rarely interact with people who care about me; These days, I feel disconnected from other people; These days, I often feel like an outsider in social gatherings). This study suggested that youth may have more difficulty understanding the negatively valanced items [36].

In the current paper we report the TB total score as outlined in the INQ-12. Scale items were rated on a Likert scale ranging from 1 = not at all true for me to 7 = very true for me. Each subscale’s total score ranged between 6 and 42. Higher scores reflected greater PB. The wording of items on the TB scale were inversely valanced compared to PB, and therefore items were recoded so that higher scores also reflected greater TB. In the current study internal consistency for PB among 12–15-year-olds was excellent (preintervention α = 0.94; postintervention α = 0.97) and 16–17-year-olds (preintervention α = 0.93; postintervention α = 0.96). The TB scales also demonstrated adequate reliability among 12–15-year-olds was (preintervention α = 0.78; postintervention α = 0.79) and 16–17-year-olds (preintervention α = 0.77; postintervention α = 0.89).

### Borderline symptoms

**McLean’s Screening Instrument for Borderline Personality Disorder (MSI-BPD; **37**)** was included in the initial assessment. This is a 10-item screening tool commonly utilised to assess symptoms of borderline personality disorder among adults. Participants indicate ‘yes’ or ‘no’ to items that assess symptoms as aligned with the categorical diagnosis of BPD. The MSI-BPD has been found to valid and reliable (α = .73) when utilised among adolescent inpatients and in this context a cut-off score of 5.5 was recommended for 12-17-year-olds [38]. In the current study, the reliability of the measure in the 12–15-year-old cohort was α = .54 and 16–17-year-old cohort was α = .62.

**Borderline Personality Features Scale for Children – 11 items (BPFS-C-11; **39**)** was utilised to assess borderline features among 12–15-year-olds in the current study. This is an 11-item assessment tool which rates items on a Likert rating scale ranging from 1= not true at all to 5 = always true. This scale has been validated among inpatient adolescents aged 14–19 years and a cut-off point of 34 is recommended. Cronbach’s alpha reported for the tool is α = 0.85 [39]. In the current study internal consistency for 12–15-year-olds was just below the acceptable level at preintervention but adequate postintervention (preintervention α = 0.66; postintervention α = 0.86).

**Borderline Symptom List – 23 and supplement (BSL-23 & BSL-supp; **40**).** Adolescents aged between 16 and 17 years completed the BSL-23 and BSL-supp. This assessment aligned with the core measure of BPD symptom severity utilised with adults in community mental health settings [22], where most of these older adolescents were receiving the intervention. The BSL-23 includes 23-items assessing borderline symptom severity on a Likert Scale ranging between 0 = not at all to 4 = strongly agree. Items are summed and then averaged to provide a total mean score between 0 and 3.

This tool also includes an additional supplementary scale (BSL-supp) which assesses the frequency of 11 self-destructive/impulsive behaviours on a Likert Scale ranging between 0 = not at all through to 4 = daily or more often resulting in a total score ranging between 0 and 44. Of particular relevance to this study are three items that assess the frequency of suicide-related behaviour ‘I told other people that I was going to kill myself’, ‘I hurt myself by cutting, burning, strangling, head banging etc’ and ‘I tried to commit suicide’.

Both assessments have demonstrated adequate reliability (BSL-23 α = 0.91; BSL-supp α = 0.77) in another sample of Australian adolescents with a diagnosis of BPD [41]. In the current study the BSL-23 internal consistency for 16–17-year-olds was excellent (preintervention α = 0.92; postintervention α = 0.97). The internal consistency for the BSL-supp demonstrated good reliability for 16–17-year-olds (preintervention α = 0.81; postintervention α = 0.86).

### Psychosocial functioning

**Work and Social Adjustment Scale – Youth (WSAS-Y; **42**)** is a brief and commonly used 5-item self-report assessment of the impact of the adolescent’s mental health difficulties on their ability to function across school/employment, everyday activities, social and personal leisure, and family/relationships. Each item is measured on a 9-point Likert scale assessing impairment ranging from 0 = not at all to 8 = severely impaired. Items are summed into a total score ranging between 0 and 40. The tool has demonstrated adequate validity, test-retest reliability, and high internal consistency (α = 0.84; 34, 35). In the current study internal consistency for 12–15-year-olds was acceptable (preintervention α = 0.77; postintervention α = 0.78) and 16–17-year-olds (preintervention α = 0.70; postintervention α = 0.87).

### Guardian reports at preintervention: 12–15 years only

Finally, guardians were asked to complete a brief assessment of younger adolescents’ borderline features and psychosocial functioning at preintervention. They completed the parent versions of the borderline features scale (BPFS-P-11; 43) and the work, social and adjustment scale (WSAS-P; 42, 44) which both had slight wording changes compared to the child version (for parental relevance) and were scored and interpreted in the same way as the child versions. In the current study internal consistency for the parent rating was good for both the BPFS-P-11 (α = 0.82) and WSAS-P (α = 0.84).

### Service outcomes

We will evaluate whether the intervention had a sustained impact on service utilisation by comparing each person’s number of ED presentations, inpatient admissions, and bed days in the 6-months before commencing Gold Card SA relative to the 6-months after their final session.

### Implementation indicators

Finally, we will report early implementation indicators captured through routinely collected data, as drawn from the broader literature [31, 45]. *Acceptability* was indicated by patient-reported ratings of intervention ‘helpfulness’ on a Likert Scale ranging from 1 = not at all helpful through to 5 = extremely helpful, after the final session. *Appropriateness* will be informed indirectly through intake diagnosis and the proportion of adolescents screening above the cut-off for BPD features. Although the intervention is flexible in the number of sessions adolescents completed (up to three), session attendance was used as dose-related *fidelity* indicator to describe the amount of intervention delivered. *Fidelity* to the model will also be informed by the number of carer sessions attended and whether adolescents high in borderline features had discharge recommendations onto structured psychological interventions in line with the NHMRC guidelines [15]. We will report the number of consumers receiving the intervention and staff and LHNs which delivered over time as indictors of *penetration* and *sustainability.*

### Statistical approach

Data were screened for missing items, accuracy, and assumptions of normality. Total scale scores for most scales in the 12–15-year-old dataset were normally distributed across each scale except for the MSI-BPD and TB at preintervention. The 16–17-year-old dataset included a number of scales that were not normally distributed. Parametric tests were used for analysis of descriptives among the younger cohort of adolescents whereas non-parametric tests were undertaken for the older adolescents. Implementation indicators are reported using descriptive data.

Patient-reported outcome measures (PROMs) were examined utilising linear mixed models (LMMs) to examine the fixed effect of time (initial assessment and postintervention) and a random effect for individual participants across each of the dependent variables. This approach has an advantage over traditional paired comparison techniques as it accounts for repeated measures, individual differences across participants, it is robust to the effects of missing data and violations to assumptions of normality [46]. Post hoc estimated marginal means were computed for each timepoint and compared using Bonferroni adjustments to control for Type 1 error. Change in PROMs pre and postintervention were estimated using Cohen’s *d*_*av*_ and interpreted as 0.2 = small, 0.5 = medium, 0.8 = large [47].

An additional analysis was conducted for the 16–17-year-old group who reported the frequency of self-destructive/impulsive behaviours pre- and postintervention. Generalised estimating equations (GEEs) were utilised to examine whether there was a significant change in the odds of respondents engaging in each of the self-destructive/impulsive behaviours in the week before their first and final sessions. To undertake this analysis responses were recoded to create a binary variable (0 = not at all; 1 = once or more). Time was entered as a fixed factor and repeated measures were specified with an autoregressive (AR 1) covariance structure. Odds ratios are reported as Exp(B).

To analyse change in service utilisation in the 6-months before the first appointment and 6-months after the final appointment we utilised negative binomial regression (NBR) given that count data were over-dispersed. The fixed effect of time (6 months pre- and postintervention) on acute mental health service utilisation variables (i.e. number of ED presentations, inpatient admissions and bed days), were expressed as an incident rate ratio (IRR).

## Results

### Participant flow

**Fig. 2:**
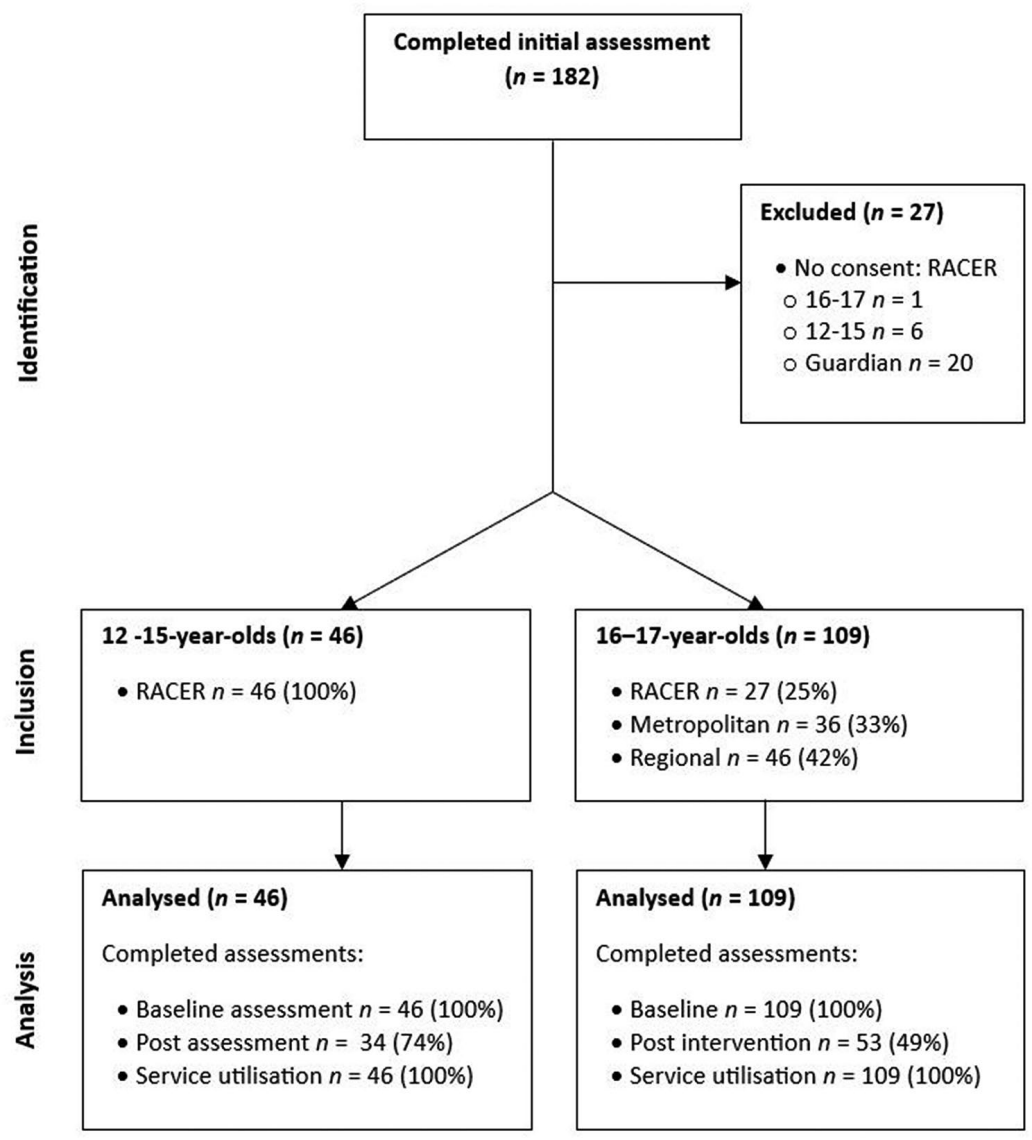
Participant flow through Gold Card SA

At least *n* = 72 adolescents aged 12–15 years were offered an initial Gold Card SA session via the RACER clinic. However, *n* = 26 were not included in the research as they or their guardian did not provide consent. One older adolescent (16–17-year-old) completed Gold Card SA via the RACER clinic but was excluded from the evaluation as they did not provide research consent. We were unable to track the total number of older adolescents (16–17-year-olds) referred to Gold Card SA within the community mental health teams as we only audited age for participants from the records of those who consented and there was not a way to separate 16-17-year-olds out from the broader group of adults referred without first identifying them. Participant flow is outlined in Fig. 2.

### Baseline descriptives

The sample aged 12–15 years were predominantly girls (*n* = 37, 80%). Guardians of children 12–15 years old were mostly mothers (*n* = 32, 70%) followed by fathers (*n* = 13, 28%), and one person did not define as either. The guardian’s ages spanned between 28 and 67 years (*M* = 45.88, *SD* = 8.16). Examining the single MSI-BPD item completed by young adolescents at preintervention ‘*Have you deliberately hurt yourself physically? How about made a suicide attempt?*’ a response of ‘*yes*’ was endorsed by *n* = 44 (96%) of this cohort. Almost all younger adolescents were referred from the WCHN ED.

Participants aged 16–17 years were predominately girls 72% with a *M*_*age*_ *=* 16.52 (*SD* = 0.50). In response the MSI-BPD item at preintervention, ‘*Have you deliberately hurt yourself physically? How about made a suicide attempt?*’ *n* = 97 (90%) responded *‘yes’*. The most frequent referral source to Gold Card SA for this cohort was the WCHN ED (45%) followed by a community source (22%) such as a general practitioner (GP), family member, school or the young person themselves. Further demographic details are highlighted in Table 1.

**Table 1:** Participant demographic characteristics split by age cohort

Characteristic		12–15 years (*n* = 46) *n* %	16–17 years (*n* = 109) *n* %
Age, *M (SD)*		14.26 (0.95)	16.52 (0.50)
Gender ^a^	Girls	37 (80%)	78 (72%)
	Boys	4 (9%)	21 (19%)
	TGNC	5 (11%)	9 (8%)
Ethnicity ^b^	Australian	36 (80%)	85 (83%)
	Aboriginal and/or Torres Strait Islander origin	2 (4%)	7 (7%)
	Other	6 (16%)	9 (9%)
Referral	ED -WCH	44 (96%)	47 (45%)
Source ^c^	ED – Other	-	6 (6%)
	Hospital/Inpatient setting	1 (2%)	15 (14%)
	Community	-	23 (22%)
	Mental Health Triage/ CMHT	1 (2%)	7 (7%)
	Not-for-profit organisation	-	6 (6%)
Number of	1	3 (7%)	18 (17%)
Sessions	2	2 (4%)	17 (16%)
	3 or more	41 (89%)	74 (68%)
Carer Session	Yes	43 (94%)	54 (51%)
Diagnosis	Intake	1 (2%)	11 (13%)
F60.31	Discharge	2 (4%)	37 (38%)

Note. TGNC = transgender and gender non-conforming; ED = emergency department; WCH = women’s and children’s hospital; CMHT = community mental health team; F60.31 emotional unstable personality disorder, borderline

^a^ 1 person missing as preferred not to disclose gender. ^b^ Missing data for two people. ^c^ Data regarding ethnicity missing for 10 people

### Patient-reported outcome measures

As indicated in Table 2, the 12–15-year-old cohort demonstrated small statistically significant reductions in PB (*d*_*av*_ = 0.31), TB (*d*_*av*_ = 0.30), and psychosocial dysfunction (*d*_*av*_ = 0.31). There was no significant reduction in borderline personality features. In contrast, we observed a large statistically significant reduction in borderline symptom severity for 16–17-year-olds who completed the intervention (*d*_*av*_ = 0.89) and a moderate reduction in PB (*d*_*av*_ = 0.51). This group also demonstrated small statistically significant reductions in psychosocial dysfunction (*d*_*av*_ = 0.31) and mean ratings of impulsive/self-destructive behaviour (*d*_*av*_ = 0.40). In contrast to the younger adolescents, there was no significant change in TB scores among 16-17-year-olds.

**Table 2:** Estimated marginal means and difference between pre- and postintervention PROMs split by age cohort

Variable	Preintervention EMM (95% CI)	Postintervention EMM (95% CI)	Mean Difference EMM (95% CI)	*p* value	Effect Size Cohen’s d_av_
**12–15 years (n = 46)**					
Perceived burdensomeness	25.67 (22.83, 28.52)	22.49 (19.27, 25.72)	-3.18 (-5.01, -1.36)	0.001	0.31
Thwarted belongingness	25.80 (23.95, 27.66)	23.88 (21.77, 25.99)	-1.92 (-3.42, -0.42)	0.01	0.30
Borderline features – children	38.26 (36.57, 39.95)	37.92 (35.55, 40.29)	-0.34 (-2.24, 1.55)	0.71	0.04
Psychosocial dysfunction	23.02 (20.41, 25.63)	20.09 (17.36, 22.81)	-2.94 (-4.98, -0.90)	0.006	0.31
**16–17 years (*n*** **= 109)**					
Perceived burdensomeness	28.26 (26.30, 30.23)	22.82 (19.89, 25.74)	-5.45 (-8.10, -2.79)	< 0.001	0.51
Thwarted belongingness	26.39 (25.00, 27.78)	24.99 (22.67, 27.31)	-1.40 (-3.76, 0.95)	0.24	0.25
Borderline symptom severity	2.73 (2.59, 2.87)	2.00 (1.73, 2.27)	-0.73 (-0.97, -0.49)	< 0.001	0.89
Impulsive/self-destructive behaviour	0.74 (0.62, 0.86)	0.51 (0.35, 0.67)	-0.24 (-0.40, -0.08)	0.004	0.40
Psychosocial dysfunction	25.69 (24.14, 27.24)	22.83 (20.35, 25.31)	-2.86 (-5.02, -0.70)	0.01	0.31

Note. EMM = estimated marginal means; CI = confidence interval

*p* < .05

### Frequency of self-destructive/impulsive behaviour

GEE analysis was undertaken to examine the frequency of self-destructive/impulsive behaviours reported by 16–17-year-olds in the first and final session. As reported in Table 3, estimated marginal means indicated that 62% of 16–17-year-olds told someone that they wanted to kill themselves in the week before their first appointment and the odds of this occurring at the end of the intervention significantly decreased by 26% (Exp(B) = 0.74, *p* < .001). Statistically significant reductions in the odds of behaviours occurring between pre- and postintervention were also noted for suicide attempts (Exp(B) = 0.75, *p* < .001), deliberate self-harm (Exp(B) = 0.86, *p* = .03), induced vomiting (Exp(B) = 0.88, *p =* .02), misuse of medication (Exp(B) = 0.89, *p* = .04), and high risk behaviours (Exp(B) = 0.86, *p* = .01).

**Table 3:** Odds ratio of 16–17-year-olds engaging in self-destructive/impulsive behaviours pre- and postintervention

Frequency of self-destructive/impulsive behaviours	Preintervention EMM (95% CI)	Postintervention EMM (95% CI)	Mean Difference (95% CI)	*p*	Exp(B)
1. I told other people that I was going to kill myself	0.62 (0.53, 0.71)	0.32 (0.20, 0.44)	0.30 (0.16, 0.44)	< 0.001	0.74
2. I tried to commit suicide	0.43 (0.33, 0.52)	0.14 (0.04, 0.24)	0.28 (0.17, 0.40)	< 0.001	0.75
3. I hurt myself by cutting, burning, strangling, head banging etc.	0.67 (0.58, 0.76)	0.52 (0.39, 0.65)	0.15 (0.01, 0.28)	0.03	0.86
4. I had episodes of binge-eating	0.54 (0.44, 0.63)	0.53 (0.41, 0.66)	0.00 (-0.12, 0.12)	0.95	1.00
5. I induced vomiting	0.33 (0.24, 0.42)	0.19 (0.09, 0.29)	0.13 (0.02, 0.24)	0.02	0.88
6. I displayed high-risk behaviour	0.35 (0.26, 0.44)	0.19 (0.09, 0.29)	0.16 (0.03, 0.28)	0.01	0.86
7. I got drunk	0.28 (0.19, 0.36)	0.25 (0.14, 0.37)	0.02 (-0.09, 0.14)	0.68	0.98
8. I took drugs	0.29 (0.20, 0.37)	0.27 (0.17, 0.37)	0.02 (-0.08, 0.11)	0.75	0.98
9. I took medication that had not been prescribed (or more than prescribed)	0.24 (0.16, 0.32)	0.13 (0.04, 0.22)	0.11 (0.01, 0.22)	0.04	0.89
10. I had outbreaks of uncontrolled anger or physically attacked others	0.43 (0.33, 0.52)	0.31 (0.19, 0.44)	0.11 (-0.03, 0.25)	0.12	0.89
11. I engaged in sexual activities that I later felt ashamed about	0.21 (0.14, 0.29)	0.17 (0.08, 0.27)	0.04 (-0.06, 0.14)	0.41	0.96

Note. Includes all patients with outcome data available for at least 1 timepoint (*N* = 108). EMM = Estimated Marginal Means; CI = Confidence Interval; Exp(B) = odds ratio. *p* < .05

### Service utilisation

We examined adolescent’s utilisation of acute public sector mental health (MH) services (i.e., number of MH-related ED presentations, inpatient admissions and bed days) in the 6-months before the initial Gold Card SA session and after their final appointment. All but *n* = 2 younger adolescents receiving the intervention had at least one ED presentation in the previous 6-months (95.7%; Range = 1–3), which was reflective of this being the key referral source to the RACER clinic. In the 6-months after participation in the intervention, only 13% of the 12–15-year-old cohort had another ED presentation. As highlighted in the results of negative binomial regression modelling outlined in Table 4, the incidence rate of MH-related ED presentations in the 6-months after the brief intervention was 90% lower relative to the 6-months beforehand (95% CI [0.05, 0.18]; *p*<.001).

**Table 4:** Mental health-related public hospital service utilisation outcomes from negative binomial regression models by age cohort

Variable	6-month preintervention EMM (95% CI)	6-month postintervention EMM (95% CI)	Incidence Ratio (95% CI)	*p* value
**12–15 years (n = 46)**				
ED presentations	1.33 (1.09, 1.61)	0.13 (0.07, 0.24)	0.10 (0.05, 0.18)	< 0.001
Inpatient Admissions	0.20 (0.10, 0.38)	0.04 (0.01, 1.8)	0.22 (0.05, 1.05)	0.06
Bed days	0.22 (0.10, 0.46)	0.07 (0.02, 0.24)	0.30 (0.07, 1.29)	0.11
**16–17 years (*n*** **= 109)**				
ED presentations	1.31 (1.08, 1.60)	0.31 (0.21, 0.46)	0.24 (0.16, 0.35)	< 0.001
Inpatient Admissions	0.65 (0.45, 0.95)	0.22 (0.13, 0.37)	0.34 (0.20, 0.57)	< 0.001
Bed days	1.72 (0.95, 3.11)	0.89 (0.48, 1.62)	0.52 (0.25, 1.06)	0.07

Note. EMM = estimated marginal means; CI = confidence interval; ED = emergency department

*p* > .05

In the 6-months before the intervention, *n* = 9 (19.6%) adolescents in the 12–15-year-old cohort had one inpatient admission spanning between 1 and 2 bed days. This reduced to only *n* = 2 adolescents (4.3%) having an inpatient admission in the 6-month postintervention period and fewer bed days. The reduction in inpatient admission incidence was not statistically significantly different between the two periods (IRR 0.22, 95% CI [0.05, 1.05), *p* = .06) which may reflect the fact that overall, there were only a small number of inpatient admissions in the 6-month preintervention period.

Examination of the 16 -17-year-old data revealed that 74.3% of the sample had one or more ED presentations in the 6-months preintervention (Range *=* 1–9) which decreased to 32.9% of 16–17-year-olds having an ED presentation in the 6-months postintervention (Rang*e* = 1–2). The incidence rate of ED presentations was 76% lower in the 6-month postintervention relative to the 6-months preintervention (95% CI [0.16, 0.35]; *p*<.001). It was also evident that *n* = 43 (39.4%) adolescents in the 16–17-year-old cohort had an inpatient admission in the 6-months preintervention (Range = 1–7) spanning between 1 and 31 bed days. The incidence rate of inpatient admissions was also 76% lower in the 6-months postintervention, relative to the 6-month preintervention period (95% CI [0.20, 0.57]; *p*<.001). The incidence of bed days was not statistically significantly different between the two periods (IRR 0.52, 95% CI [0.25, 1.06), *p* = .07).

### Implementation indicators

#### Acceptability

Overall, *n* = 33 young adolescents (12–15-years-old) completed the postintervention assessment at their final appointment and 63.6% rated the support and strategies offered through Gold Card SA as ‘moderately to extremely helpful’. Most participants in 16-17-year-old cohort who completed the evaluation at the final session rated the intervention as ‘moderately to extremely helpful’ (*n* = 44, 86%).

#### Appropriateness

Findings from demographic characteristics such as diagnosis at intake and the MSI-BPD cut-off scores provide an indication of whether the intervention was delivered to an appropriate cohort of adolescents. Among 12–15-year-olds the primary intake diagnosis was suicidal ideation (*n* = 18; 40%), following by mental health condition not otherwise specified (*n* = 7, 16%), poisoning (*n* = 5, 11%) and only one person had a BPD diagnosis (2%). Preintervention *n* = 39 (85%) rated above the cut-off on the MSI-BPD and *n* = 36 (78%) on the BPFS-C-11. In comparison, guardian ratings on the BPFS-P-11 indicated that *n* = 26 (57%) of young adolescents met the cut-off score.

The primary diagnosis for older adolescents at intake was most often suicidal ideation (*n* = 31, 35%), followed by mental health condition not otherwise specified (*n* = 10, 18%), and BPD (*n* = 11, 13%). Examination of the MSI-BPD total scores exceeding 5.5 points revealed that 91% (*n* = 97) of older adolescents scored above the cut-off.

#### Fidelity

Gold Card SA offers adolescents up to 3 individual sessions which can be considered a dose-related fidelity indicator. In the young adolescent cohort, *n* = 31 (67%) attended three sessions while an additional *n* = 10 (22%) were offered a fourth session. Among older adolescents, *n* = 67 (62%) attended three sessions, while *n* = 7 (6%) attended more than this amount. There was a high level of involvement in the carer session in the younger adolescent cohort (94%). Approximately, 51% of older adolescents had someone attend the carer session (mostly parents (95%), grandparents (4%), and in one case a partner (2%)).

We reviewed discharge recommendations split by young people who had a diagnosis of BPD at discharge, and those that did not have a diagnosis, but had either low or high borderline features based on their preintervention MSI-BPD scores (see Table 5). Among adolescents with a diagnosis of BPD or high borderline features (*n* = 117) only a small proportion were referred onto Dialectical Behaviour Therapy (DBT; *n* = 3; 3%) or the statewide specialist service for people diagnosed with BPD (*n* = 13; 11%).

**Table 5:** Discharge recommendations split by diagnosis of borderline personality disorder or features (high/low)

Discharge recommendations	BPD diagnosis		No diagnosis – High features		No diagnosis – Low features		Total sample (*n* = 130)
	12–15 (*n* = 2)	16–17 (*n* = 30)	12–15 (*n* = 37)	16–17 (*n* = 48)	12–15 (*n* = 7)	16–17 (*n* = 6)	
General practitioner	1 (50%)	17 (57%)	29 (78%)	38 (79%)	6 (86%)	2 (33%)	93 (72%)
Psychologist/Private therapy practice	1 (50%)	5 (17%)	10 (27%)	8 (17%)	3 (43%)	1 (17%)	28 (22%)
Psychiatrist	0 (0%)	3 (10%)	2 (5%)	5 (10%)	1 (14%)	1 (17%)	12 (9%)
Not-for-profit organisation (i.e., headspace, Baptist care)	1 (50%)	7 (23%)	12 (32%)	10 (21%)	2 (29%)	1 (17%)	33 (25%)
Community mental health team	1 (50%)	3 (10%)	11 (30%)	6 (13%)	0 (0%)	1 (17%)	22 (17%)
Borderline personality disorder collaborative (e.g., short-term group, website)	0 (0%)	5 (17%)	0 (0%)	8 (17%)	0 (0%)	0 (0%)	13 (10%)
Dialectical behaviour therapy	0 (0%)	1 (3%)	0 (0%)	2 (4%)	0 (0%)	0 (0%)	3 (2%)
Other (e.g. eating disorder service, inpatient ward)	0 (0%)	2 (7%)	3 (8%)	4 (8%)	0 (0%)	1 (17%)	10 (8%)

*Note. n* = 25 missing discharge recommendations; BPD = borderline personality disorder

#### Penetration and sustainability

**Fig. 3:**
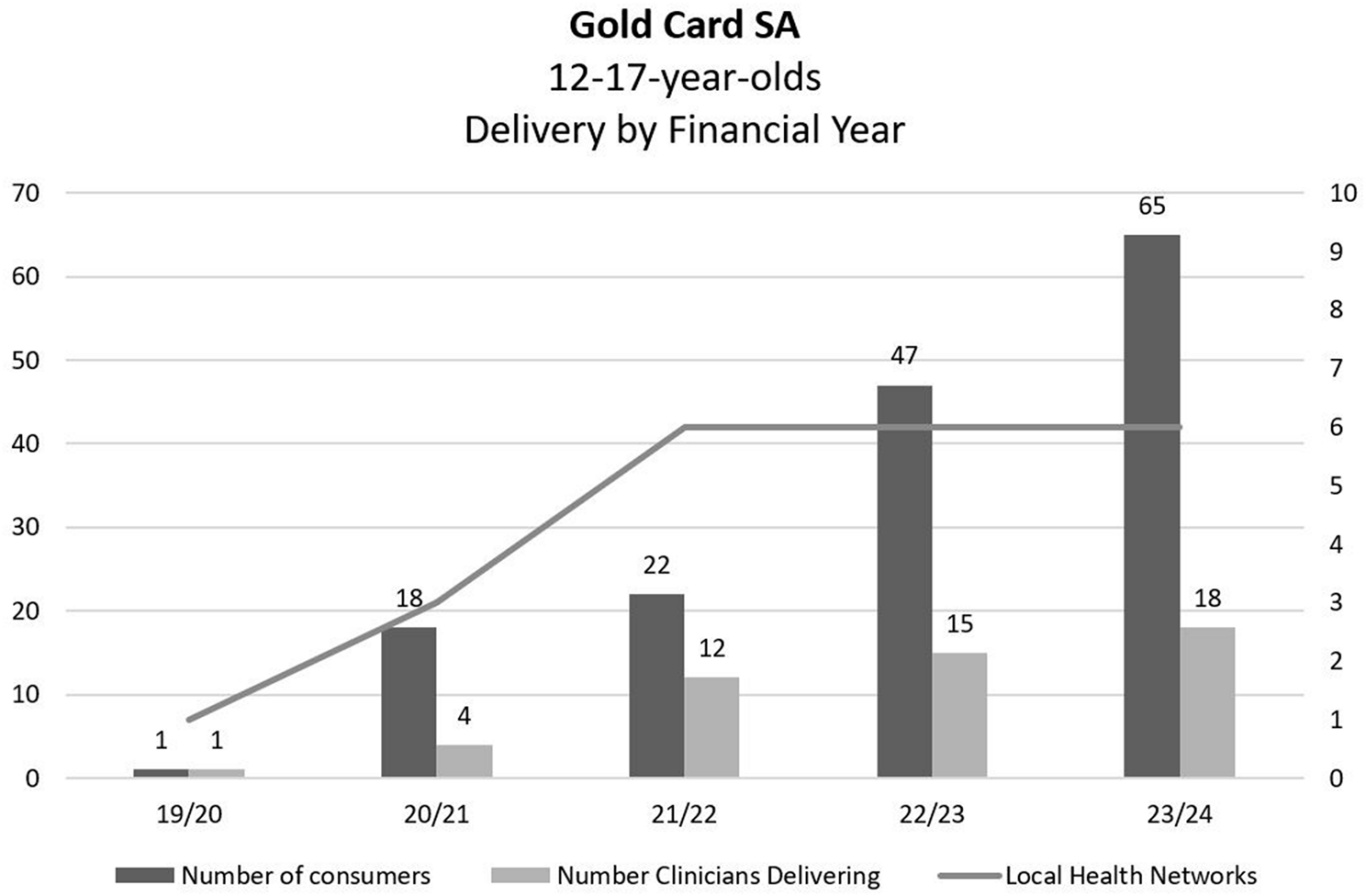
Gold Card SA delivery to adolescents by number of staff across local health networks each financial year

The intervention was delivered by *n* = 37 clinicians comprised of social workers (*n* = 15; 41%), nurses (*n* = 14; 38%), occupational therapists (*n* = 5; 14%), psychologists (*n* = 2, 5%), and a psychiatry registrar (3%). Over half of the clinicians offered the intervention to one adolescent (*n* = 21, 56%), whereas 35% of clinicians (*n* = 13) saw more than one adolescent but less than 10, and *n* = 3 (8%) clinicians delivered the intervention to more than 20 adolescents each. The intervention was delivered in eight of the 10 LHNs in the state, primarily the Women and Children’s Health Network (*n* = 73, 47%), then regional and remote sites (*n* = 46; 30%), and metropolitan community mental health teams (*n* = 36, 23%). Figure 3. demonstrates growth in the number of consumers receiving Gold Card SA and staff delivering between 2019 and 2024.

## Discussion

This study examined the large-scale implementation of a brief intervention for adolescents in an acute mental health crisis. Suicidal thoughts and behaviours were high at intake across both younger (12–15-year-old; 96%) and older adolescents (16–17-year-olds; 90%). The majority of younger (85%) and older adolescents (91%) presenting in crisis had MSI-BPD cut-off scores indicative of high borderline symptoms. Younger adolescents also completed the BPFS-C-11 and 75% met the cut-off indicative of elevated borderline features. In comparison, guardian ratings on the BPFS-P-11 were more conservative with 57% rated as exceeding the cut-off score. These findings align with other studies which suggest that personality disorder features may be a risk factor for adolescents presenting in crisis to acute mental health services [4, 5] and therefore warranting targeted intervention.

Effectiveness outcomes derived from PROMs embedded within the intervention indicated a large statistically significant reduction in borderline symptoms severity for older adolescents, comparable to studies of adults who received the intervention [22, 24]. Similar to adult outcomes, older adolescents demonstrated moderate reduction in perceived burdensomeness (PB; 22). In contrast, they reported relatively smaller reductions in self-destructive/impulsive behaviour and psychosocial dysfunction. Thwarted belonging (TB) did not significantly reduce which may be indicative of the developmental stage in which young people are ‘trying to find their tribe’. They are moving away from their parents and are more reliant on relationships with peers which may open more opportunities for feeling left out [48].

Younger adolescents did not demonstrate a significant reduction in borderline personality features. They reported significant yet small reductions in PB, TB, and psychosocial dysfunction. These findings should be interpreted whilst taking into consideration developmental differences between younger and older adolescents. Research into the effectiveness of early intervention for BPD among adolescents has shown that older adolescents tend to report greater reductions in psychopathology than younger adolescents [49]. In general, the period of early adolescence is associated with a large dip in subjective wellbeing [50] compared to mid-adolescence which may also confound our findings. It has also been suggested that the effectiveness of early intervention for younger adolescents may become evident through preventing the trajectory of increased BPD symptoms with age [51].

The incidence of ED presentations significantly reduced in the 6-months postintervention, relative to the 6-months preintervention across both cohorts of adolescents. There was also a significantly lower incidence of inpatient admissions among older adolescents in the 6-month postintervention period. Whilst the incidence of admissions was not significantly lower in the postintervention period among younger adolescents, this may be influenced by the small number of admissions at 6-months preintervention, with only *n* = 2 participants having an admission in the 6-months postintervention.

Our findings should be considered in the context of early implementation indicators. Older adolescents (86%) who completed the intervention rated it as ‘moderately to extremely helpful’ suggesting acceptability. However, fewer younger adolescents (63%) provided this rating suggesting lower acceptability. In terms of dosage, many adolescents (75%) attended all three sessions with some attending an additional session. This is consistent with research demonstrating that adolescents attend an average of 4.2 sessions in real-world clinical settings [52]. In the current study, carer session engagement was highest among younger adolescents (94%) relative to older adolescents (51%), yet still exceeded rates reported for adults [22, 24], reflective of the developmental stage of participants.

This brief intervention was implemented within a broader model of stepped care in which it is recommended that adolescents high in borderline features are referred onto evidence-based therapies where appropriate [15, 34]. Review of discharge recommendations suggested that the majority of youth were referred back to their treating GP, followed by a not-for-profit service or a private psychologist/therapy practice. Only a small proportion of those who had a diagnosis of BPD/high in borderline features were referred onto an evidence-based therapy for BPD such as DBT or a specialist BPD service. Additional investigation is needed to better understand the availability, appropriateness and accessibility of evidence-based treatments in the community for adolescents with high borderline features.

Finally, we reported early indicators of intervention penetration and sustainability which showed a growing number of clinicians delivering and the intervention over time, and increased numbers of adolescents receiving it. However, it was apparent that more than half of the clinicians only provided the intervention to one adolescent, suggesting potential implementation barriers which need further exploration. In stating this, the spread of the intervention over most LHNs in the state was encouraging with almost all delivering the intervention.

### Strengths and limitations

These findings need to be interpreted with caution as we did not have a control group, nor were people randomised to conditions, which would have helped to minimise bias, account for potential regression to the mean, and identify natural remission post-crisis. Missing data at the second time point means that some of the findings, including ratings of acceptability, will be biased towards those who completed all sessions. The intervention was delivered predominately to Australians, with a smaller proportion identifying as from a different ethnic background (10%) or Aboriginal and/or Torres Strait Islander (5%). Given the suicide rate of Aboriginal and Torres Traits Islander people is significantly greater than that of non-indigenous Australians, it is important that suicide prevention services are holistic, culturally sensitive, and co-designed [53]. Further exploration is needed into the cultural appropriateness of this intervention and/or access to alternative pathways where needed.

In the present study, service utilisation outcomes were only examined 6-months prior to the first appointment and 6-months after the last session. This extended upon the 3-month range reported by Teasdale and colleagues [28] but longer follow-up periods such as 12-months or more may reveal patterns of behaviour over time. Only presentations related to mental health were included in the service utilisation counts which may underrepresent attendances related to a physical cause (e.g., stomach-aches) which may also be intertwined with mental health.

Our evaluation was weighted towards PROMS and service outcomes, and did not include a priori measurement of key implementation outcomes such as costs nor adoption or feasibility relative to broader system capacity. For example, we were unable to estimate whether the reduction in acute service utilisation resulted in cost-savings that outweighed those incurred through the training and multi-site implementation support provided [54]. Additionally, we did not collect stakeholder ratings of acceptability and appropriateness from guardians or clinicians which may have helped us better understand treatment response in the younger adolescents and why over half of the clinicians delivered the intervention to only one adolescent.

However, these limitations are bolstered by strengths of this study. Our evaluation has high ecological validity. We demonstrated the potential to deliver this brief intervention to young people in crisis in a real-world public mental health service. Youth in this context presented with high acuity and complexity. Implementation spanned multiple sites and settings with penetration into rural and remote areas. As anticipated, we observed high levels of borderline personality features among this cohort of adolescents in mental health crisis supporting the rationale for tailored care pathways in this setting.

### Implications for policy and practice

The findings of this study have a number of practical implications for policy and practice. The inclusion of a brief personality dysfunction screener in acute mental health settings can identify adolescents that may benefit from being stepped into other interventions in the community [5]. However, this also means that there needs to be accessible and tailored interventions for youth presenting with borderline personality features/diagnosis in the community. As noted previously, young people presenting to primary mental health services in Australia (i.e., headspace) with BPD diagnosis or traits were offered CBT, supportive counselling, and IPT, in primary youth mental health services with limited effectiveness [14]. Wood and colleagues [55] also reported on the local implementation of a self-harm intervention and a DBT-A group program in a public sector youth mental health setting. Implementation barriers such as high staff turnover made it difficult to sustain the program. Given recent meta-analysis of the literature demonstrated the effectiveness of DBT as a treatment for adolescents with a diagnosis of BPD or subthreshold symptoms [16] studies that take into consideration both implementation and effectiveness outcomes may be particularly helpful in real-world settings.

## Conclusion

Gold Card SA is a promising brief crisis intervention for adolescents presenting with suicide-related behaviours, even in the presence of elevated borderline personality features. Across cohorts, we noted a significant reduction in the incidence of ED service use in the 6-months postintervention, relative to beforehand. Older adolescents reported a large significant reduction in borderline symptom severity and reduced odds of engaging in suicide-related behaviours. Further consideration is needed for how systems can increase access to evidence-based interventions for younger adolescents presenting with borderline personality features to facilitate early intervention.

## Supplementary Information

Below is the link to the electronic supplementary material.

Supplementary Material 1

## Data Availability

The data generated or analysed during the study are not publicly available due to privacy and ethical reasons but are available from the corresponding author at reasonable request.
