# Supplementary material for: Implementation and outcomes of a brief crisis intervention for adolescents with borderline personality features: a pre-post observational study
Source: BMC Psychiatry. 2026 Feb 6;26:237. doi: 10.1186/s12888-026-07871-y (PMC12983568; doi:10.1186/s12888-026-07871-y)
Supplement: Supplementary file 1 — Supplementary Material 1 [file 12888_2026_7871_MOESM1_ESM.pdf]

Supplementary Table S1.

Statewide Implementation Strategy for Gold Card SA

Implementation strategy	Mechanisms
1. Leadership endorsement	• Statewide advisory committee.<br>• Memorandums of administration agreements between statewide service and all LHNs.<br>• LHNs to self-nominate.<br>• Executive committee established to oversee implementation.
2. Tailor to the environment	• Site implementation committee established to lead implementation.<br>• Development of local governance processes.
3. Engage the local mental health team	• Identify local 'champions' within the team.<br>• Statewide service clinician linked with implementing team in LHN to support building team readiness.
4. Training	• Participation in 1-day '*Foundational skills for working with people with BPD*' training, followed by 1-day '*Gold Card SA*' training.
5. Supervision and mentoring	• Statewide service clinician was linked with implementing teams to provide regular peer supervision and mentoring.
6. Evaluation	• PROMs embedded within the intervention delivery.<br>• Use and interpretation of PROMs included within training.<br>• Electronic data collection, automated scoring and reports delivered to clinicians in real-time to inform assessment and intervention.<br>• PROMs utilised within local team's clinical review processes.<br>• Regular reporting mechanisms (e.g., providing teams with aggregate outcomes).

Note. LHN = local health network; PROM = patient-reported outcome measure
